# Right Time, Right Place: The Four-Dimensional Pattern of Fly Neuron Development

**DOI:** 10.1371/journal.pbio.1000369

**Published:** 2010-05-11

**Authors:** Richard Robinson

**Affiliations:** Freelance Science Writer, Sherborn, Massachusetts, United States of America

**Figure pbio-1000369-g001:**
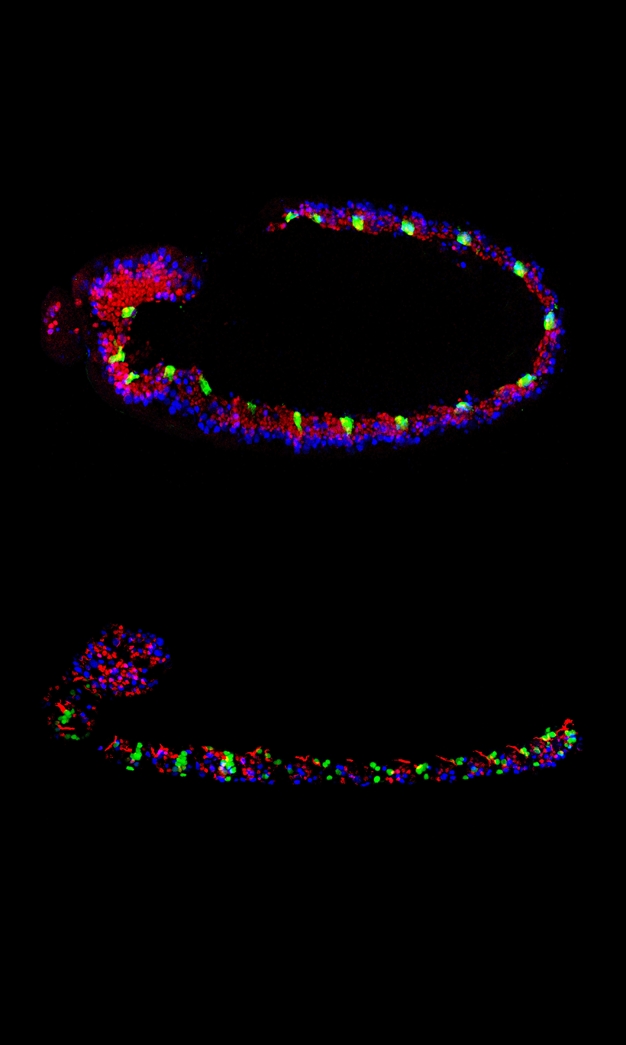
The nervous system of the fly develops from neuroblasts arranged into segments along the larval body (shown with a green fluorescent marker). **The differentiation of specific nerve cell types in the thoracic segments results from spatial and temporal control of neuroblast lineages via an interplay of Hox, Pbx/Meis, and temporal genes.**

[Fig pbio-1000369-g001]Embryonic development is a four-dimensional process: in order to create an organism, the right genes must be expressed at the right time and in the right place. But while the overall strategy of embryogenesis is clear—successive waves of gene expression give rise to a pattern of transcription factors varying in time and space, driving cell differentiation—relatively little is known about the four-dimensional details of this process for most cell types. In a new study in this issue of *PLoS Biology*, Daniel Karlsson, Magnus Baumgardt, and Stefan Thor elucidate some of those details in the developing nervous system of the fly and show that major differences between body regions can arise from simple changes in gene expression.

In the fly, the nervous system develops from progenitor cells called neuroblasts, arranged into 18 segments that are lined up down the length of the larval body. In the thoracic segments, located about a third of the way down from the head, certain neuroblasts generate four neurons called the Ap (for “apterous”) cluster, distinguished from other neurons by the expression of a specific set of transcription factors.

Surprisingly, equivalent neuroblasts are present not only in thoracic segments but also in abdominal and brain segments. So, why are Ap cluster neurons formed only in thoracic segments?

In previous work, the authors showed that Ap cluster neurons form at the end of a long series of neuroblast cell divisions and only in the presence of specific molecular signals. Here, they found that neuroblast cells in the abdomen stopped dividing before reaching the stage that would create Ap clusters. A group of powerful pattern-generating genes called the bithorax complex, which is able to turn cell division off, is expressed at the proper time in the abdomen, and so, the authors tested its role in terminating abdominal neuroblast division. They found that switching the complex off by mutation allowed cell division to proceed, and Ap clusters to form, in abdominal segments.

Another key player in preventing Ap cluster formation in the abdomen was the gene *castor*. *castor* is one of a sequence of “time window” genes that turn on and then off during development. Some events, including Ap cluster formation, are possible only when the Castor window “opens,” which occurs later in development, after abdominal neuroblasts have already stopped dividing.

In the thorax, the bithorax complex is inactive. But several other genes appeared to play critical roles. One, called *homothorax*, was present in high concentrations in thoracic segments, and its mutation prevented Ap cluster formation, suggesting its presence was critical for forming Ap clusters. With the Castor window open, *homothorax* could promote expression of another gene, *collier*, which triggers expression of the markers that define Ap cluster neurons. Two other genes, including *Antennapedia*, were also needed to get full collier expression.

In the most anterior segments, which make up the fly brain, neuroblast division continued long enough to make Ap clusters, but nonetheless the clusters didn't form. The reason, the authors found, was that these segments lacked *Antennapedia*, as well as another critical time-window gene acting in the thorax, called *grainyhead*. When they induced brain segments to express both *Antennapedia* and *grainyhead*, the segments developed Ap clusters, just like thoracic segments did. When they simultaneously expressed these genes and blocked the bithorax complex, almost every segment, from abdomen to brain, developed Ap clusters as if they were all thoracic segments.

While the details are complicated, the underlying logic that emerges from this study is unexpectedly simple. The thoracic segment development pathway can be considered the default pattern, and the two regions that are “not thorax”—abdomen and brain—employ two entirely distinc strategies for becoming different. In the abdomen, progenitor cells are turned off early, whereas in the brain, specific promoting factors are withheld. These results are likely to lead to further insights into general patterns of fly development and, since many of these genes are shared by vertebrates, will likely be more widely applicable as well.

**Karlsson D, Baumgardt M, Thor S (2010) Segment-Specific Neuronal Sub-Type Specification by the Integration of Anteroposterior and Temporal Cues. doi:10.1371/journal.pbio.1000368**

